# Canine sperm motility is associated with telomere shortening and changes in expression of shelterin genes

**DOI:** 10.1186/s12917-023-03795-x

**Published:** 2023-11-10

**Authors:** Hossein Hassanpour, Pezhman Mirshokraei, Marzieh Salehpour, Khadije Amiri, Parvin Ghareghani, Leila Nasiri

**Affiliations:** 1 Department of Gametes and Cloning, Research Institute of Animal Embryo Technology, Shahrekord University, Shahrekord, Iran; 2 Department of Clinical Sciences, Faculty of Veterinary Medicine, Ferdowsi University of Mashhad, Mashhad, Iran; 3 Department of Biology, Faculty of Basic Sciences, Shahed University, Tehran, Iran

**Keywords:** Telomere, Shelterin, Kinematic parameters, Sperm, Gene expression

## Abstract

**Background:**

Motion quality is a critical property for essential functions. Several endogenous and exogenous factors are involved in sperm motility. Here, we measured the relative telomere length and evaluated the gene expression of its binding-proteins, shelterin complex (*TRF1, TRF2, RAP1, POT1, TIN2*, and *TPP1*) in sperm of dogs using relative quantitative real-time PCR. We compared them between two sperm subpopulations with poor and good motion qualities (separated by swim-up method). Telomere shortening and alterations of shelterin gene expression result from ROS, genotoxic insults, and genetic predisposition.

**Results:**

Sperm kinematic parameters were measured in two subpopulations and then telomeric index of each parameter was calculated. Telomeric index for linearity, VSL, VCL, STR, BCF, and ALH were significantly higher in sperms with good motion quality than in sperms with poor quality. We demonstrated that poor motion quality is associated with shorter telomere, higher expression of *TRF2, POT1*, and *TIN2* genes, and lower expression of the *RAP1* gene in dog sperm. The levels of TRF1 and TPP1 gene expression remained consistent despite variations in sperm quality and telomere length.

**Conclusion:**

Data provided evidence that there are considerable changes in gene expression of many shelterin components (*TRF2, TIN2, POT1*and *RAP1*) associated with shortening telomere in the spermatozoa with poor motion quality. Possibly, the poor motion quality is the result of defects in the shelterin complex and telomere length. Our data suggests a new approach in the semen assessment and etiologic investigations of subfertility or infertility in male animals.

## Background

Fertility problems sometimes occur in male animals during their reproductive activation. In the canine breeding industry, infertility results in substantial financial losses for dog owners, and a complete breeding soundness evaluation is necessary for detection of the causes [1]. A critical factor in sperm assessment is kinematic parameters that are essential for the spermatozoa to migrate toward the fertilization site in the oviduct and to pass across the zona pellucida both in vivo and in vitro. Thus it has been noticed as a critical factor in determining fertilization rate [2]. Potent sperm motility facilitates fertilization via better passing of sperm across the cumulus cell, corona radiate, and finally, the zona pellucida. It has been confirmed that sperm motility is strongly influenced by reactive oxygen species (ROS) in seminal plasma causing mitochondrial disorders [3, 4], structural malformation in the flagella [5], and DNA damage [6]. Numerous studies have demonstrated a correlation between telomere shortening and the occurrence of sperm DNA damage, hence implicating that this phenomenon may contribute to male infertility [7].

Telomere structure consists of short, tandem repeats of DNA sequence that cover linear chromosome ends by linking members of the shelterin protein complex to provide protective telomere loops. An inadequate number of telomere repeats causes chromosome uncapping, cell aging, and death [8]. During meiosis of germ cells, telomeres play vital roles in the protection of chromosome ends from nucleolytic degradation/fusion and in tethering chromosomes together to the nuclear envelope. It is also necessary for prosperous synapsis between homologs as well as for the proper resolution of recombination events [9].

Telomere shortening results from ROS, genotoxic insults, and genetic predisposition. ROS destroys telomeres via oxidizing its guanine-rich parts; thereby promoting a DNA damage response, which leads to the excision of telomere repeats [10] and then biological aging [11]. It has been determined that gene mutations involving telomerase activity or telomere stability lead to the occurrence of many clinical disorders (named telomeropathies) in germline cells. These disorders often originate from short or malfunction telomeres [12].

Shelterin is a protein complex known to protect telomeres. Six proteins, telomere repeat binding factor 1 and 2 (TRF1 / TRF2), repressor/activator protein 1 (RAP1), TRF-interacting nuclear protein 2 (TIN2), telomere protection protein 1 (TPP1), and protection of telomeres 1 (POT1), form the shelterin complex, that is a fundamental part of telomeres [13]. The TRF1/TRF2 homodimer joins double-stranded telomeric repeats while POT1 connects to the single-stranded telomeric 3′ overhang. TIN2 links, through protein interactions, TRF1, TRF2, and TPP1. TPP1 joins, in addition, to POT1, therefore recruiting POT1 also to the double-stranded segment of telomeres [14]. RAP1 relates to telomeres through interaction with TRF2. POT1 also binds directly to TRF2. The shelterin complex plays a vital role in both telomere stability and cell signaling reactions. Disturbing expression levels of shelterin members mainly affect the telomere length [14].

In this study, we separated spermatozoa from dog semen by swim-up method and classified them into good and poor motion qualities, namely the top-layer (TL) and bottom-layer (BL) sperm group respectively. Their relative telomere length and expression of shelterin genes (*TRF1, TRF2, RAP1, POT1, TIN2*, and *TPP1*) were measured, and compared between two sperm subpopulations. This study aimed to investigate the involvement of telomere length and shelterin proteins in sperm motion quality.

## Results

### Sperm kinematic parameters

Table 1 indicates the kinematic parameters for sperm subpopulations of top and bottom layers after swim-up. Sperm parameters of total motility, progressive motility, linearity, VSL, VCL, STR, BCF, and ALH were significantly more in the TL-sperm group than in the BL-sperm group. In contrast, the parameter of non-motility was less.

**Table 1:** Comparison of kinematic parameters between sperm subpopulations of top and bottom layers after swim-up

Parameters	Sperms of top layer	Sperms of bottom layer	P value
Total motility (%)	73.7 ± 3.83	40.7 ± 4.02*	< 0.001
Progressive motility (%)	51.9 ± 4.86	11.6 ± 2.28*	< 0.001
Non-motility (%)	28.3 ± 3.83	59.3 ± 4.11*	< 0.001
VSL (μm/s)	24.9 ± 3.54	4.9 ± 0.94*	< 0.001
VCL (μm/s)	66.7 ± 6.46	39.7 ± 2.97*	0.002
Linearity (VSL/VCL) (%)	30.3 ± 2.31	11.2 ± 1.08*	< 0.001
STR (VSL/VAP) (%)	56.5 ± 2.88	30.2 ± 1.58*	< 0.001
BCF (Hz)	5.2 ± 0.69	3.1 ± 0.51*	0.004
ALH (μm)	4.1 ± 0.24	3.1 ± 0.20*	< 0.001

Values are means ± standard error of the mean (SEM); VCL, curvilinear velocity; VSL, straight line velocity; ALH, lateral head displacement; STR, straightness of trajectory, BCF, beat cross frequency. * Significant difference between two groups in each row (*P* < 0.05)

### Sperm telomere length and telomere index of kinematic parameters

**Fig. 1:**
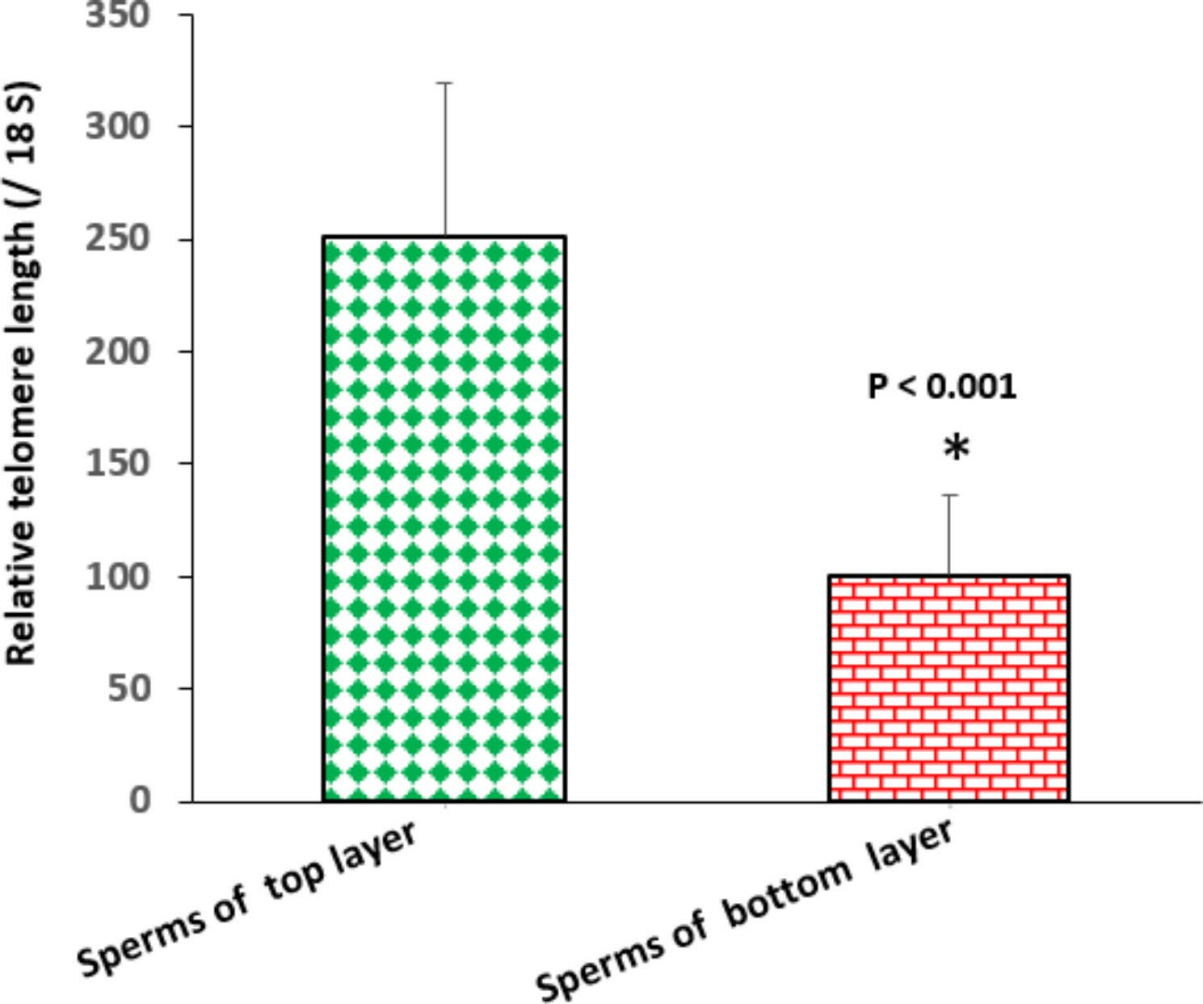
Comparison of relative telomere length between sperm subpopulations of top (green diamond) and bottom (red brick) layers after swim-up. Data are given as mean ± standard error of the mean (SEM). * Significant difference between two groups (*P* < 0.001)

Figure 1. shows the relative amounts of telomere length for sperm subpopulations of top and bottom layers after swim-up. Mean telomere length was significantly more in the TL-sperm group than in the BL-sperm group (P < 0.05).

**Fig. 2:**
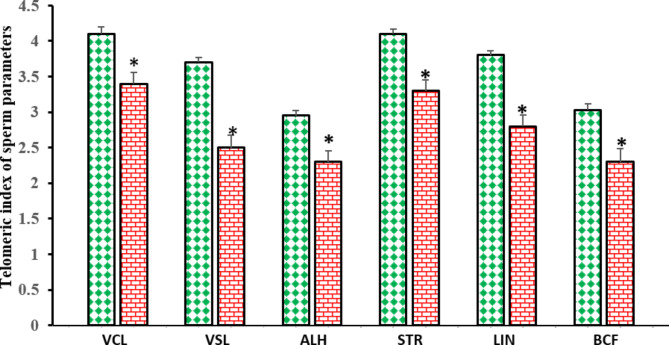
Telomeric index of sperm kinematic parameters [log (parameter × telomere length)]. VCL, curvilinear velocity; VSL, *straight line* velocity; ALH, lateral head displacement; STR, straightness of trajectory, LIN, linearity; BCF, beat cross frequency. Data are given as mean ± standard error of the mean (SEM). *Significant difference (*P* < 0.05) between sperm subpopulations of top (green diamond) and bottom (red brick) layers after swim-up

Figure 2. displays the telomeric index of sperm kinematic parameters for sperm subpopulations of top and bottom layers after swim-up. Telomeric index for linearity, VSL, VCL, STR, BCF, and ALH were significantly more in the TL-sperm group than the BL-sperm group (P < 0.05).

### Relative expression of shelterin genes

**Fig. 3:**
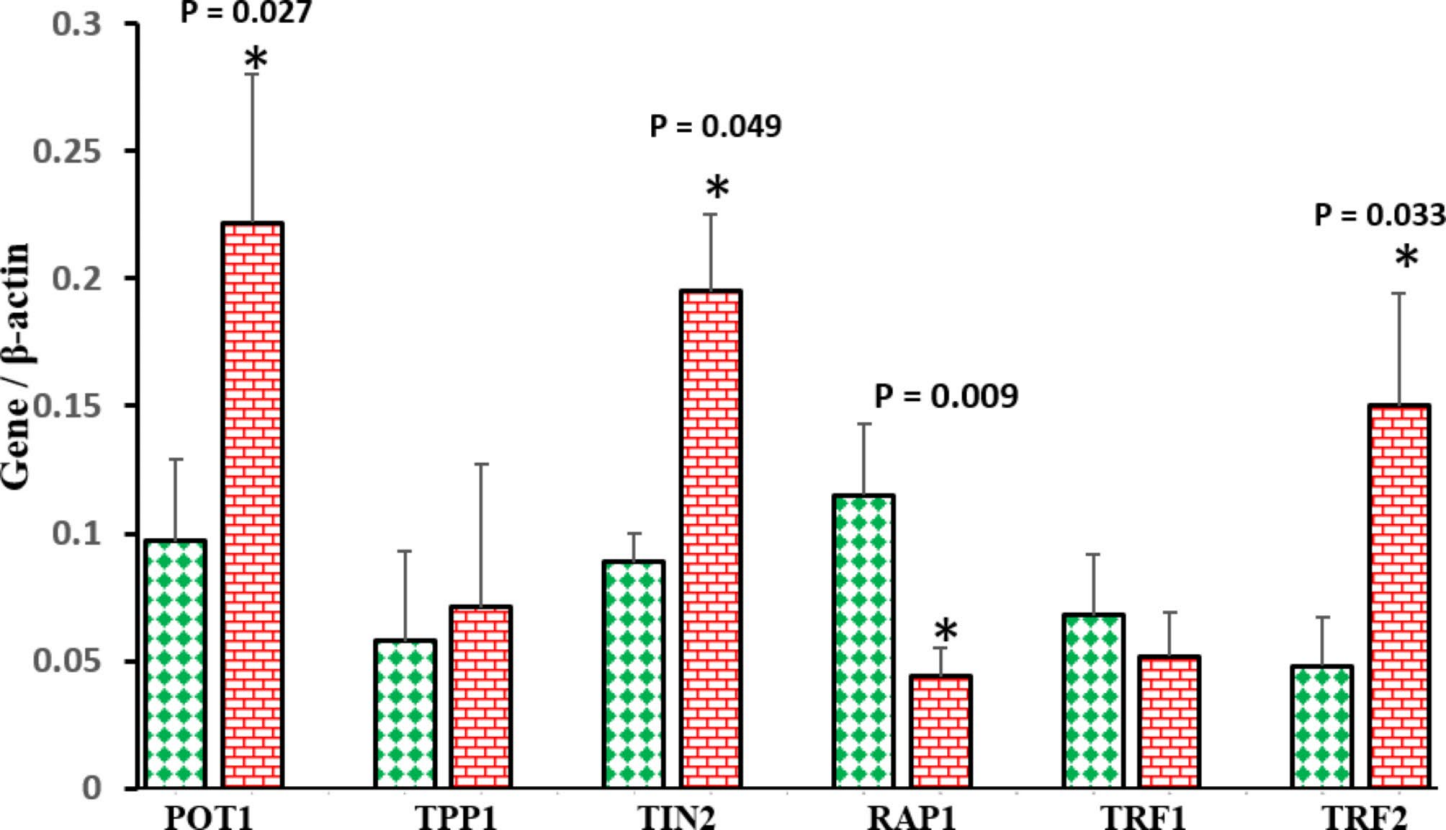
Comparison of relative expression of shelterin genes between sperm subpopulations of top (green diamond) and bottom (red brick) layers after swim-up. Data are given as mean ± standard error of the mean (SEM). * Significant difference between two groups (*P* < 0.05)

Figure 3. indicates the relative expression of shelterin genes in the TL- and BL-sperm subpopulations obtained after swim-up. The relative expression of the *RAP1* gene was significantly more in the TL-sperm group than the BL-sperm group, while the relative expression of *POT1, TIN2*, and *TRF2* genes was significantly less in the TL-sperm group than the BL-sperm group (P < 0.05). The gene expression of *TPP1* and *TRF1* did not change between the two groups of sperm subpopulations.

## Discussion

Motility is a complex physiological property of spermatozoa, which is controlled by several extrinsic and intrinsic factors during spermatogenesis and sperm transit from seminiferous tubules to the fertilization site (oviduct). Any alteration of these factors leads to various severity of stress and disruption in the normal motility development in sperm. The external and internal stressors play a critical role in the induction of oxidative stress, chromosome mutations, immunogenetic disorders, and apoptosis. They affect the activity of enzymatic and non-enzymatic responses that lead to a downgrading in sperm motion quality [15]. Because of susceptibility of the sperm nuclear chromatin concentration period to oxidants and lack of the DNA repair mechanism in the sperm cells, chromosomal damages are expectable during spermatogenesis and sperm maturation. Damages such as base modifications, frameshifts, deletions, cross-linking, DNA strand breaks, rearrangement of chromosomes, and telomere shortening have been reported [16, 17]. In this study, there was a considerably longer telomere size in the high motile sperm subpopulation separated by swim-up method. In this regard, Santiso et al. [18] reported that following swim-up of human semen, the sperm subpopulation of the top layer has a longer telomere length and lower DNA fragmentation. Our finding is also in agreement with other studies suggesting the assessment of sperm telomere length as an index for chromatin integrity and infertility [19, 20].

In this study, the relative expression of six shelterin genes (i.e., *TRF1, TRF2, RAP1, POT1, TIN2*, and *TPP1*) was evaluated in the sperm subpopulations with good (longer telomere) and poor (shorter telomere) motion qualities, but only four of those considerably changed. *TRF2, POT1*, and *TIN2* were increased, and *RAP1* decreased in the subpopulation with poor motion quality (shorter telomere).

*TRF2* is the core component of shelterin which plays a prominent role in telomere stability and maintenance of normal cell physiology. *TRF2* folds telomeres into loops to inhibit undue DNA damage response, avoids the ataxia telangiectasiamutated kinase signaling and telomere nonhomologous end joining (NHEJ), and promotes telomere replication. It has been confirmed that *TRF2* protein mutation or abnormal expression leads to T‑loop destruction, loss of DNA end protection, end‑to‑end fusion, chromosomal inconstancy, cell aging, apoptosis, or anomalous transformation [21]. Many previous studies evaluated the interaction of *TRF2* overexpression and its function in mammalian cells. Smogorzewska et al. [22], Richter et al. [23] and Nera et al. [24] indicated that following the induction of high levels of TRF2 in cell lines, progressive shortening of telomere length resulted. On the other hand, Matsutani et al. [25] suggested that gastric carcinoma tissue with short telomere and chromosomal instability overexpresses the *TRF2* to create the structure of D-loop and T-loop and protect the telomere ends. Muñoz et al. [26] and Benetti et al. [27] also showed that the telomere was degraded in *TRF2-*overexpressed mouse models and XPF nuclease over-activity led to this degradation, causing premature aging and increased cancer. The mentioned studies confirm our data that there is an association between the telomere shortening and *TRF2* overexpression, although comparing the function of sperm and cancer cells may not be a sensible approach, and more detailed studies on sperm are needed for confirmation.

*TIN2*, as an adaptor protein plays a linking role in the shelterin complex. This protein participates in the regulation of DNA damage response. Its mutations are associated with telomeric DNA impairment, telomere instability related to telomerase activity, and premature aging [28]. Chen et al. [29] found that *TIN2* is a regulator of metabolism that controls mitochondrial oxidative phosphorylation. Hu et al. [30] determined that the overexpression of *TIN2* and *TRF2* proteins counteract the effects of the TERT protein in gastric cancer tissue and leads to further telomere shortening. This data is consistent with our finding of *TIN2* in spermatozoa with shorter telomeres, but further study on sperm is required to validate this result, as mentioned, it does not seem reasonable to compare the function of sperm and cancer cells. It is perhaps that overexpression of *TIN2* in association with *TRF2* is a vital factor in the telomere shortening of low motile spermatozoa. On the other hand, a high level of *TIN2* may affect sperm metabolism by disrupting the function of mitochondria; of course, new research is necessary to confirm the latter issue.

*POT1*, as a critical component of the shelterin complex, regulates telomere length and telomere capping. This protein protects the chromosome ends from recombination, chromosome instability, and abnormal segregation [31]. It has been demonstrated that *POT1* represses the efficiency of NHEJ, repairs DNA double-strand breaks, and progresses NHEJ fidelity [32]. D Gomez, et al. [33] reported that *POT1* overexpression in cell lines improves both telomere and G-overhang length. It should be noticed that the mentioned studies evaluated *POT1* at the level of cell translation while our results were at the level of *POT1* transcription that is earlier to the translation. Therefore, in the spermatozoa with shorter telomere, the increase of *POT1* transcript may be the evidence of a compensatory effect to produce more *POT1* protein for improving the telomere. However, further study is needed to evaluate factors involving *POT1* translation and to measure the level of *POT1* protein in these cells.

*RAP1* is another component of the shelterin complex that protects the telomere ends using the activation of different DNA repair mechanisms. It has several roles in controlling the gene expression, specific signaling pathways (e.g., NF-κB pathway), and metabolism [28]. It has been revealed that overexpression [34] or depletion of the *RAP1* [35] may cause telomere shortening, leading to cell aging. However, the here observed association of lower RAP1 in low motile spermatozoa with shorter telomeres, may be in agreement with many previous studies [34, 35].

In addition to the direct effects of shelterin proteins in regulating the telomere length, they also act indirectly via controlling the telomerase activity [28]. It is possible that alterations of shelterin components like *TRF2, TIN2, POT1*, and *RAP1* as observed in this study, change the telomere length via effect in the activation of telomerase enzyme.

## Conclusions

we provided evidence that there are considerable changes in gene expression of many shelterin components (*TRF2, TIN2, POT1* and *RAP1*) associated with telomere shortening in the spermatozoa with poor motion quality. Possibly, a decrease in kinematic characteristics could be the result of defects in the shelterin complex and telomere length.

## Methods

All used chemicals were purchased from Sigma Chemical Co. (Louis, MO).

### Animals, collection and swim-up of ejaculated sperm, and CASA analysis

Semen samples of good quality were collected from ten male crossbred dogs (2–4 years old). Dogs were cared at the Faculty of Veterinary Medicine (Ferdowsi University of Mashhad) and housed in pens with ample runs. They were fed well twice a day and were given access to water ad libitum. The project underwent ethical review and was approved by the local Ethics Committee of Shahrekord University and Ferdowsi University of Mashhad (IR.SKU.REC.1400.076). Before semen collection, a complete breeding soundness examination was performed consisting of a history, physical examination (general and andrological), semen evaluation, and testing for *Brucella* canis. In clinical andrological examination, the genital organs were checked by palpation or sonography [36]. An expert operator collected the ejaculated semen (sperm-rich fraction) from each dog using a funnel collecting vial and manual massage of the penis, and then immediately transported it to the laboratory. All semen samples had a volume of about 0.8-3 ml (sperm-rich fraction), and color of pearly white or translucent. Sperm morphology was evaluated by microscopic analysis (1000×) of a slide stained with eosin-nigrosin. All semen samples expressed a minimum of 70% normal sperm morphology. Semen samples were initially evaluated by computer-assisted sperm analysis (HFT CASA, Hoshmand Fanavar Tehran, Iran). Semen samples had a concentration of about 100–200 × 10^6^ spermatozoa/ml. Only samples that had progressive motility more than 60% were used in this study. Equal volumes of semen and a primary sperm diluent without antibiotic (consisting of 69.4 mM fructose, 249.8 mM Tris, and 80.9 mM citric acid) [37] were mixed, then centrifuged at 700 x g for 5 min. The supernatant was discarded, and the pellet containing sperm was resuspended in 100 μl diluent. For swim-up, this sperm sample (100 μl) was gently over-layered with pre-warmed 2 ml diluent in a conical tube which was sealed, inclined at 45 degrees and incubated for 15 min at 37 °C in an atmosphere containing 5% CO2. After this time, sterile Pasteur pipettes were used to gently collect 100 μl of the diluent containing sperm from each top (from its upper part) and bottom layer to new microtubes. CASA analysis was repeated for separated sperm samples from the top layer (as TL-sperm) and bottom layer (as BL-sperm). Finally, these sperm samples were stored at -70 °C until subsequent DNA and RNA extractions.

CASA system was set according to [38], and then analysis was done using Mackler chambers (20 μm depth). In this system, the following kinematic parameters were measured: total motility (%), progressive motility (%) and non-motility (%), straight-line velocity (VSL, μm/s) is the average velocity measured in a straight line from the beginning to the end of one track; the curvilinear velocity (VCL, μm/s) is the average velocity measured over the actual point to point track followed by the cell, the amplitude of lateral head displacement (ALH, μm), is the degree of lateral displacement of the sperm head’s centroid around its average path; the beat cross frequency (BCF, Hz), is the frequency at which the sperm cell’s head crosses the sperm cell’s average pathway; the linearity (LIN, %) is the linearity of a curvilinear path; the straightness (STR, %) is the proximity of the cell’s pathway to a straight line [38].

### Genomic DNA extraction and telomere size analysis by quantitative real-time PCR

Genomic DNA was extracted directly from sperm cells (samples of TL-sperm and BL-sperm) using a High yield DNA Purification Kit (DNP™ Kit, SinaClon BioScience, Karaj, Iran) according to the manufacturer’s instructions.

**Fig. 4:**
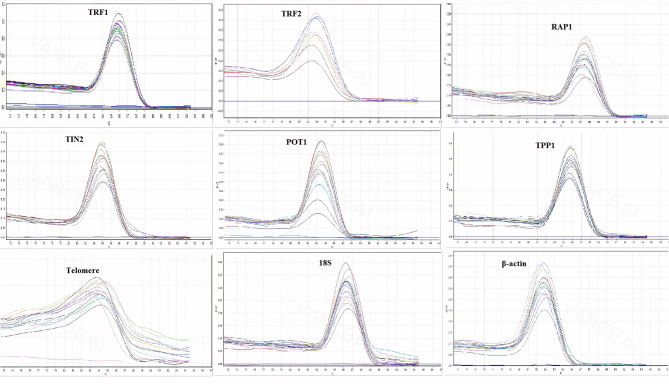
Specificity of real time PCR amplification. Melting curves (dissociation curves) of the 7 target genes and 2 reference genes (β-actin and 18 S) amplicons after the real time PCR reactions, all showing one peak. X-axis (horizontal): temperature (C); Y-axis (vertical): negative derivative of fluorescence over temperature (dF/dT)

To measure the relative telomere length of sperm, relative quantitative real-time PCR (RT-qPCR) was performed using a SYBR® Premix Ex Taq™ II (Tli RNaseH Plus) kit (Takara Bio Inc., Japan). The telomere primers used were Tel1 (5′-GGTTTTTGAGGGTGAGGGTGAGGGTGAGGGTGAGGGT-3′) and Tel 2 (5′-TCCCGACTATCCCTATCCCTATCCCTATCCCTATCCCTA-3′) [39]. The 18 S ribosomal RNA (18 S) gene (167 bp amplicon) was chosen as the reference gene [40]. The primer sequences used were 18 S-F (5′- GGCATTCGTATTGCGCCG-3′) and 18 S-R (5′-ATCGCCAGTCGGCATCGT-3′). The melt curves of both telomere and 18 S following amplification showed a single peak (Fig. 4), evidence of specific amplification. The amplification was done in a final volume of 10 μl for both telomere and 18 S. A volume of 9 ng was used in each reaction. The concentration of each primer was 250 nM. Amplifications were performed in triplicate for each sample in a Rotor-Gene 6000 thermocycler (Qiagen, Australia). The PCR program for telomere was 95 °C for 10 min and 20 cycles of 95 °C for 15 s and 54 °C for 2 min. The PCR program for 18 S was 95 °C for 10 min and 35 cycles of 95 °C for 20 s, 60 °C for 20 s and 72 °C for 20 s. A no-template control reaction was run to ensure no contamination. The threshold cycle number (Ct) of PCR and mean efficiency values (E) for telomere (T) and 18 S (S) were determined using LinRegPCR software (2012.0, Amsterdam, Netherlands). Relative telomere length (T/S ratio) was calculated according to Pfaffl [41] and Näslund et al. [42]. In this method, the level of telomere length relative to 18 S was counted for each sample using the following formula:

$${\rm{E}}{\,_{{\rm{18S}}}}{\,^{{\rm{(Ct}}\,{\rm{sample)}}}}\,{\rm{/}}\,{\rm{E}}{\,_{{\rm{telomere}}}}{\,^{{\rm{(Ct}}\,{\rm{sample)}}}}.$$

In this study, to involve telomere length value in comparing sperm kinematic parameters between two experimental groups, an index was designed as a log (kinematic parameter × telomere length).

### RNA extraction, cDNA synthesis, and mRNAs assay by RT-qPCR

Total RNA of sperm cells was extracted with RNX-Plus solution (Sinaclon Bioscience, Karaj, Iran) and the acid guanidinium thiocyanate-phenol-chloroform single-step method according to Bahadoran et al. [43] and Kadivar et al. [44]. The obtained RNA pellet was resuspended in 20 μl DEPC-treated water and then treated with RNAase-free DNAase (SinaClon) to clean contaminating genomic DNA. The quality and integrity of RNA samples were evaluated by spectrophotometry and agarose gel electrophoresis. Only RNA samples representing an A260/A280 ratio of 1.8–2.2 were suitable for cDNA synthesis. The synthesis of cDNA was done using PrimeScript™ RT reagent Kit (Takara) according to its manufacturer’s instructions. The yielding cDNA was stored at − 20 °C until RT-qPCR [45].

To determine the possible changes in the transcriptional levels of shelterin genes (*TRF1, TRF2, RAP1, POT1, TIN2*, and *TPP1*) in two groups of TL- and BL-sperm samples, relative RT-qPCR was performed using PCR kit as mentioned above. To normalize the input load of cDNA and to quantify the relative target gene expression, β-actin was used as a stable control gene [46]. The used specific primers of genes are represented in Table 2. The PCR for each sample was performed in three replicates. 10 ng cDNA and 400 nM of each specific primer were used in a total volume of 10 μl. The program of PCR amplification was as 94 °C for 10 min, then 40–45 cycles of 94 °C for 20 s, 58–62 °C for 20 s, and 72 °C for 20 s. No-template and no-reverse transcriptase controls were used in each PCR reaction. Data of the threshold cycle numbers and mean efficiency values were recorded and calculated using LinRegPCR software. Then relative gene expression (target / β-actin) was calculated according to the Pfaffl method [41, 47].

**Table 2:** Primer used for quantitative real time PCR analysis of canine mRNAs

Target	Primer sequence (5ˊ- 3ˊ)	PCR product	Accession number
TRF1	GACCAACATGGCGGAAAGCG	120 bp	XM_038441383.1
	TGCTCCTGGTCATCTCTCGG		
TRF2	CGCGCATCGAAGAAGGAGAG	136 bp	XM_038667203.1
	CTGGATTCGACCACTGCCTC		
POT1	CCGGGGAATCAGAGTCTTGC	132 bp	XM_038686254.1
	CGAAGGCTGTCCTCCTGTTC		
TIN2	CCCCACAGTCATGCTGTTCC	102 bp	XM_038673212.1
	GGATCTGCCATGTGCATCCC		
TPP1	CAGACGCTCTCTCAAGTCCG	120 bp	XM_038667327.1
	CGTCAGATGTGTCAGGGACG		
RAP1	TAGCTATGGCGGAGGCGATG	107 bp	XM_038666994.1
	CCGCACGTAGAAGGACATGG		
β-actin	GGAGCGAGCATCCCCAAAGTTC	163 bp	NM_001195845.3
	GCCCTTCTCTGCAGGGAGAAAC		

### Statistical analysis

Data are given as mean ± standard error of the mean (SEM). To check the normality of data, Kolmogorov–Smirnov test was performed. Differences between mean values of the two TL- and BL-sperm groups were analyzed using the independent Student’s t-test in SPSS 26.0 software (IBM-SPSS, Inc, Chicago, IL, USA). Differences were considered significant at *P* < 0.05.

## Data Availability

All data generated or analyzed during this study are included in this published article.
